# Adults with autism spectrum conditions experience increased levels of anomalous perception

**DOI:** 10.1371/journal.pone.0177804

**Published:** 2017-05-18

**Authors:** Elizabeth Milne, Abigail Dickinson, Richard Smith

**Affiliations:** 1 Sheffield Autism Research Lab, Department of Psychology, The University of Sheffield, Sheffield, South Yorkshire, United Kingdom; 2 Sheffield Adult Autism and Neurodevelopmental Service, Sheffield Health and Social Care NHS Trust, Sheffield, South Yorkshire, United Kingdom; Universitatsklinikum Tubingen, GERMANY

## Abstract

Autism spectrum condition (ASC) is characterised by differences in social interaction and behavioural inflexibility. In addition to these core symptoms, atypical sensory responses are prevalent in the ASC phenotype. Here we investigated anomalous perception, i.e. hallucinatory and/or out of body experiences in adults with ASC. Thirty participants with an ASC diagnosis and thirty neurotypical controls completed the Cardiff Anomalous Perception Scale (CAPS) and the Social Responsiveness Scale (SRS-2). The CAPS is a 32-item questionnaire that asks participants to indicate whether or not they experience a range of anomalous and out of body experiences, and to rate how intrusive and distressing these experiences are. The SRS-2 asks participants to rate the extent to which they identify with a series of 65 statements that describe behaviours associated with the autism phenotype. We found that total CAPS score was significantly higher in the participants with ASC (mean = 14.8, S.D. = 7.9) than the participants without ASC (mean = 3.6, S.D. = 4.1). In addition, the frequency of anomalous perception, the level of distraction and the level of distress associated with the experience were significantly increased in participants with ASC. Importantly, both the frequency of anomalous perceptual experiences and the level of distress caused by anomalous perception in this sample of adults with ASC were very similar to that reported previously in a sample of non-autistic participants who were being treated in hospital for a current psychotic episode. These data indicate that anomalous perceptual experiences are common in adults with ASC and are associated with a high level of distress. The origins of anomalous perception in ASC and the implication of this phenomenon are considered.

## Introduction

Autism spectrum condition (ASC) is a pervasive developmental disorder that is diagnosed on the basis of impairments in social interaction and communication, in the presence of restricted interests and repetitive behaviours [1]. In addition to these primary symptoms, sensory issues occur frequently in ASC and there is now a wealth of empirical evidence which suggests that the majority of individuals with ASC demonstrate some form of atypical sensory behaviour. For example, one large study which reported data obtained from the short sensory profile from 291 families found that 95% of children with ASC demonstrated abnormal sensory behaviours [2]. In addition, a growing number of qualitative studies highlight sensory issues as one of the key themes of living with ASC [3]. Taking a grounded theory approach, Smith and Sharp [4] investigated the way in which atypical perception affects the lives of adults with ASC and identified heightened senses and sensory stress as commonly experienced phenomena.

The term ‘atypical perception’ is frequently used to describe sensory issues in people with ASC. It describes the fact that many people with ASC have different sensory experiences compared with people without ASC. These experiences are generally defined as hyper- or hypo-responsivity to sensory stimuli, and/or alterations in the way in which sensory input is modulated and can manifest in atypical sensory behaviours such as fascination with certain sensory experiences, highly tuned senses and / or intense dislike of some sensory stimuli. A recent study that investigated the extent to which sensory issues are correlated with self-reported autistic traits in the general population found that autistic traits are also related to anomalous perceptual experiences [5]. ‘Anomalous perception’ is distinct from atypical perception as defined above, and is the term given to perceptual and hallucinatory experiences of the type that are commonly associated with psychosis, such as hearing voices, experiencing perceptual distortions, and having ‘out-of-body’ experiences.

Anomalous perception is common in a number of psychiatric conditions including schizophrenia and psychosis spectrum disorders; it also occurs to a lesser degree in the non-clinical population [6, 7]. Individual variability in cortical excitability and individual variability in autistic-traits have, in two separate studies, been found to be associated with anomalous perception in the general population [5, 8]. For example, individuals who have had out of body experiences in the absence of any clinical diagnosis show increased sensitivity to high contrast black and white stripes—a phenomenon known as ‘pattern-glare’ that is considered to reflect increased cortical excitability [9]. Furthermore, in a large sample of neurotypical participants, scores on a the Autism-Spectrum Quotient [10] and scores on the Cardiff Anomalous Perceptions Scale (CAPS, [11]) were found to be moderately but significantly correlated [5]. Although this study included some individuals with a diagnosis of ASC no comparison of the extent to which anomalous perception occurred in the participants with and without a diagnosis of ASC was given [5]. Therefore it remains unclear whether or not anomalous perception occurs more frequently in individuals with ASC compared to those without. The aim of the study presented here was to measure anomalous perception in individuals with ASC and to compare this with a well-matched control group. A secondary aim was to investigate the effect of anomalous perception on adults with ASC by measuring the levels of distress and intrusion associated with anomalous perceptual experiences.

In line with previous work [5], the CAPS [11] was used here to investigate anomalous perception. There are a number of benefits of using this scale to measure anomalous perception in ASC. For example, the language used in the questionnaire does not assume that experiences present in a certain way (e.g. as “strange” or “unusual”), and the scale deliberately excludes items that address more general aspects of psychosis such as experiences related to thought broadcast and depersonalisation. Furthermore, in addition to measuring the number of anomalous perceptual experiences endorsed, the CAPS provides a dimensional measurement of the perceived frequency of each experience as well as the extent to which each experience is perceived as being distressing and intrusive. It is therefore well-suited to probe the effect that anomalous perceptual experiences can have.

In the development of the CAPS, nine categories of anomalous experience were defined and used to generate question items. These were: (i) changes in levels of sensory intensity, e.g. “Do you ever notice that sounds are much louder than they normally would be?”; (ii) having a non-shared sensory experience, e.g. “Do you ever experience smells or odours that people next to you seem unaware of?”; (iii) inherently unusual or distorted sensory experience, e.g. “Do you ever notice that food or drink seems to have an unusual taste?”; (iv) sensory experience from an unexplained source, e.g. “Do you ever feel that someone is touching you, but when you look nobody is there?”; (v) distortion of form of own body and of external world, e.g. “Do you ever have the sensation that your limbs might not be your own or might not be properly connected to your body?; (vi) verbal hallucinations, e.g. “Do you ever hear voices commenting on what you are thinking or doing?”; (vii) sensory flooding, e.g. “Do you ever have difficulty distinguishing one sensation from another?”; (viii) thought echo and hearing thoughts out loud, e.g. “Do you ever hear your own thoughts repeated or echoed?” and (ix) experiences associated with temporal lobe disturbance, e.g. “Do you ever have the feeling of being uplifted, as if driving or rolling over a road while sitting quietly?” Principal component analysis revealed three components within the scale which the authors labelled temporal lobe experience, chemosensation and clinical psychosis [11]. The temporal lobe experience component consists of items that are commonly reported in pre-seizure aura-type experiences by patients with temporal-lobe epilepsy [12] and in reduced form by the normal population [13]. The chemosensation component consists largely of items related to olfactory and gustatory experiences, and the clinical psychosis component consists of first-rank symptoms usually associated with clinical psychosis.

On the basis of previous work showing a correlation between anomalous perception and autistic traits [5] we predicted that anomalous perception would occur more frequently in individuals with ASC than those without. We did not have any specific hypothesis regarding any of the sub-components of the scale and whether they would be disproportionately represented in ASC, nor the extent to which anomalous perception may be perceived as distressing or intrusive by individuals with ASC.

## Materials and methods

### Participants

Participants included 30 individuals with ASC (7 females) and 30 neurotypical (non-ASC) volunteers (11 females). The mean age of the participants with ASC was 32.65 (range, 19:1 to 68.0) and the mean age of the participants without ASC was 35.37 (range, 19.0–70.9). There was no significant difference in the ages of the two groups (see Table 1). All participants in the ASC sample had received an independent diagnosis of an Autism Spectrum Disorder from an experienced clinician prior to being recruited into the study. Specific given diagnoses were: Asperger’s syndrome (N = 24); autism (N = 5); and atypical autism (N = 1). Five participants with ASC had an additional diagnosis of Attention Deficit Hyperactivity Disorder (ADHD), one person in the control group also had ADHD. Two of the participants with ASC had epilepsy and four of the participants with ASC had depression. CAPS scores for these individuals were unremarkable within the ASC sample (see Results), therefore scores from these participants were not excluded from the analysis. Prior or current diagnosis of a psychotic disorder (e.g. psychosis, schizophrenia, bipolar disorder) was an exclusion criterion of the study therefore no participants in either group had a diagnosis of this type. Participants were recruited from our existing database of research volunteers. This database consists of members of the local community who live independently, have IQ scores within the normal range and have the capacity to consent to take part in research. The age at which participants with ASC received their diagnoses varied, with 40% of the sample receiving their diagnosis before the age of 16, and 60% receiving their diagnosis after the age of 16.

**Table 1:** Participant characteristics and questionnaire scores.

	ASC Group		Non-ASC Group		*p*
	Mean	SD	Mean	SD	
Age in years	32.65	13.63	35.37	14.60	.46^a^
SRS T-score	71.80	10.63	49.27	8.36	<.001 ^b^
CAPS total score	14.80	7.94	3.63	4.11	<.001 ^b^

^a^ Given by independent samples t-test

^b^ Given by Mann-Whitney U test

### Stimuli and apparatus

Two questionnaires were administered during this study: the CAPS [11] and the revised Social Responsiveness Scale—Revised (SRS-2) [14]. The SRS-2 was included in order to (i) identify whether or not all of the participants with ASC obtained scores above the identified cut-off indicating clinically significant difficulties in reciprocal social behaviour and (ii) to measure the severity of these difficulties. Both questionnaires were completed on-line in participants’ own homes, and both questionnaires were completed in one session. The study received ethical approval from the department of Psychology ethics sub-committee. Participants were required to provide consent to take part in the study before any part of the questionnaires was presented. Consent was gained by asking participants to confirm that they had read the participant information sheet about the study and that they agreed to take part in the research. Participants were reminded that they could withdraw from the study at any time by closing the browser prior to submitting their responses. Responses were recorded between 10^th^ August 2014 and 17^th^ November 2015.

#### The Cardiff Anomalous Perceptions Scale (CAPS)

The CAPS is a 32-item self-report questionnaire that requires respondents to indicate via a ‘yes’ or ‘no’ response whether they experience particular anomalous perceptions. Instructions for completing the questionnaire were given as described by Bell et al. [11]. Participants were instructed to discount any anomalous perceptual experiences that may have occurred as a consequence of taking recreational drugs or other medication. For each endorsed item, a further three questions were presented that asked participants to rate, via a 5-point Likert scale, the frequency of the experience, and the extent to which the experience is i) distressing, and ii) intrusive.

#### The Social Responsiveness Scale—Revised (SRS-2)

The SRS-2 is a 65 item questionnaire which rates the occurrence and severity of traits and behaviours associated with ASC [14]. Participants are required to indicate, via a 4-point Likert scale, the extent to which they endorse descriptions of behaviours and traits associated with the autism phenotype, for example “I avoid eye-contact or am told that I have unusual eye contact”. Higher scores indicate a greater severity of social impairment and ASC symptomology. A T-score of 60 or above indicates clinically significant difficulties in reciprocal social behaviour [14].

## Results

Mean SRS-2 T-scores and mean CAPS scores are given in Table 1. In the non-ASC group, both SRS T-score and CAPS total score violated assumptions of normality (SRS T-score skewness = 2.07, kurtosis = 6.24; CAPS score skewness = 1.49, kurtosis = 2.52. For comparison, these values in the ASC group were: SRS T-score skewness = -.59, kurtosis = -.14; CAPS score skewness = .11, kurtosis = -.61). Therefore, where required, results from non-parametric analyses are reported below.

As expected, SRS T-scores were significantly higher in the participants with ASC than the participants without ASC, *U* = 54, *p* <.001. Four participants in the non-ASC group obtained SRS-2 T-scores of 60 or above, and four participants in the ASC group obtained SRS-2 T-scores below 60. All analyses reported below were repeated after excluding these eight participants and no result was altered. Therefore the results reported below are based on the full sample of sixty participants. The decision not to exclude participants on the basis of whether or not SRS-2 T scores correctly discriminated diagnosis status was driven by the fact that the SRS-2 is not designed for use as a diagnostic instrument and our grouping criterion was presence or absence of a clinical diagnosis of an autism spectrum condition. Nevertheless it is important to note that our sample of participants contained some individuals who were likely to be near the boundary for diagnosis.

**Fig 1:**
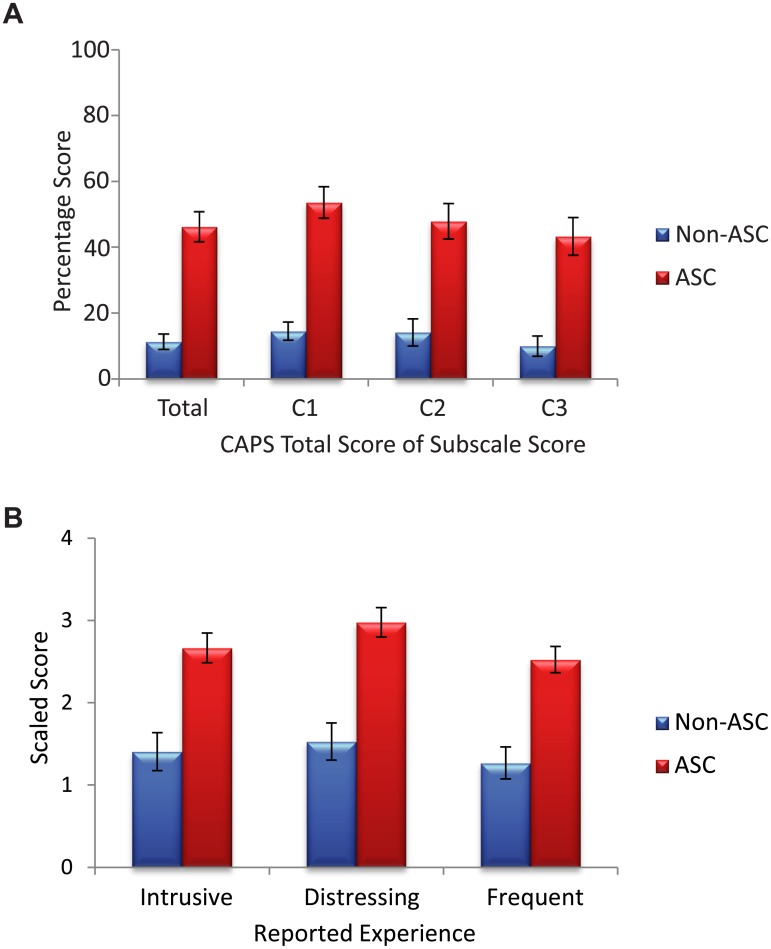
CAPS scores for the participants with and without ASC. Error bars = +/- 1 standard error. (A) shows the percentage of items endorsed for the whole scale (total), for the temporal lobe experience component (C1), for the chemosensation component (C2) and for the clinical psychosis component (C3). (B) shows the scaled level of intrusion, level of distress, and the frequency of anomalous perception reported by each group.

The following variables were calculated from the CAPS: total score (given as a percentage), and total percentage score on each of the three sub-components (‘temporal lobe experience’, ‘chemosensation’ and ‘clinical psychosis’). The latter three variables were computed by adding the number of endorsed items that were identified as loading onto these components (see 11), and dividing by the maximum component score. For ease of comparison with the total score, these variables are also given as a percentage (i.e. x 100). Eleven items were used to calculate the temporal lobe component (items 26, 4, 32, 10, 12, 24, 2, 1, 16, 27, and 6); eight items were used to calculate the chemosensation component (30, 18, 29, 21, 14, 25, 20, and 8); and four items were used to calculate the clinical psychosis component (7, 11, 3 and 31). The scores of these three components from both groups of participants are shown in Fig 1A.

Three additional variables were calculated, reflecting level of distress, level of intrusiveness, and the frequency of anomalous perception. These variables were computed separately by adding the rating for distress, intrusiveness and frequency for each endorsed item and then dividing by the total number of items endorsed. In other words, levels of distress, intrusiveness and frequency were scaled by the number of anomalous perceptual experiences reported. These data are shown in Fig 1B.

As can be seen in Fig 1A total CAPS score was significantly higher in the participants with ASC compared to the participants without ASC, *U* = 96, *p* <.001. Scores on each of the three sub-components of the CAPS were also significantly higher in the participants with ASC: temporal lobe experience *U* = 101.5; *p* <.001; chemosensation, *U* = 162; *p* <.001; clinical psychosis, *U* = 165, *p* <.001. A repeated measures ANOVA with within-subject factor of CAPS component (temporal lobe experience, chemosensation and clinical psychosis) and a between-subjects factor of group showed a main effect of group, F(1,58) = 43.16, p <.001 and a main effect of CAPS component, F(1.63, 94.28) = 3.56, p <.05. This main effect was driven by the fact that scores on the temporal lobe experience component were higher than scores on the clinical psychosis component (t = 2.39, p <.01). The interaction between group and CAPS component was not significant. Note that the CAPS scores of the individuals who had ASC and either epilepsy or depression were unremarkable compared with the rest of the sample with ASC: both participants with epilepsy scored slightly below the mean CAPS score of the ASC group, and the CAPS scores of the participants with depression ranged from 4 to 29.

Level of distress associated with anomalous perception (*U* = 144.5, *p* <.001), intrusiveness of anomalous perception (*U* = 173.5, *p* <.001) and frequency of anomalous perception (*U* = 167, *p* <.001) were also significantly higher in the participants with ASC than in those without. Note that these scores were scaled by the number of items endorsed so the increased scores in the ASC group do not simply reflect increased occurrence of anomalous perception. Rather, these data illustrate that when anomalous perceptual experience occurs, it is experienced as being more distressing, more intrusive/distracting, and also more frequent in individuals with ASC than in those without.

**Fig 2:**
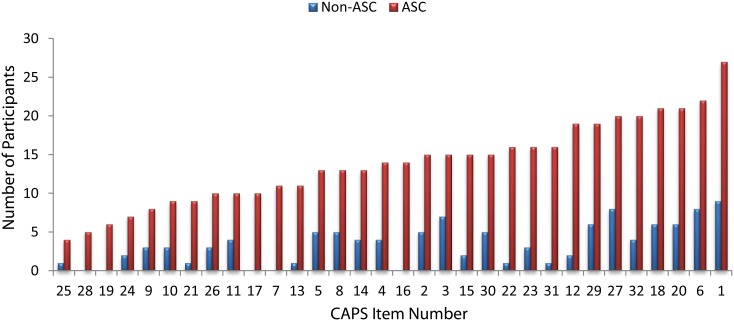
Number of endorsements given to each CAPS item by the participants with and without ASC. For ease of viewing, items have been ordered in terms of increasing numbers of participants with ASC who endorsed the item.

Some of the CAPS items, specifically those that come under category one—“changes in levels of sensory intensity”, describe sensory hypersensitivity which, as discussed above, is an established symptom of ASC. In order to investigate whether increased CAPS scores in the ASC group were being driven by a subset of questions that probed sensory hypersensitivity, we recalculated total CAPS score and associated levels of distress, intrusion and frequency after excluding items from category one (specifically, items 1, 18, 20, 21 and 23). Total CAPS score remained significantly higher in the participants with ASC, *U* = 101, *p* <.001 even after these items were excluded. Scaled scores indicating level of distress, intrusiveness and frequency were also recalculated after excluding these items and also remained significantly higher in the participants with ASC, *U* = 138, 123 and 152.5 respectively, all *p* <.001. To further check whether particular items were driving the increased CAPS score, we generated a chart to show the number of people from each group who endorsed each item. This chart, shown in Fig 2, demonstrates that every CAPS item was endorsed by more participants with ASC than without ASC, and that the increased scores in the ASC group are not being artificially inflated by a small number of items.

**Fig 3:**
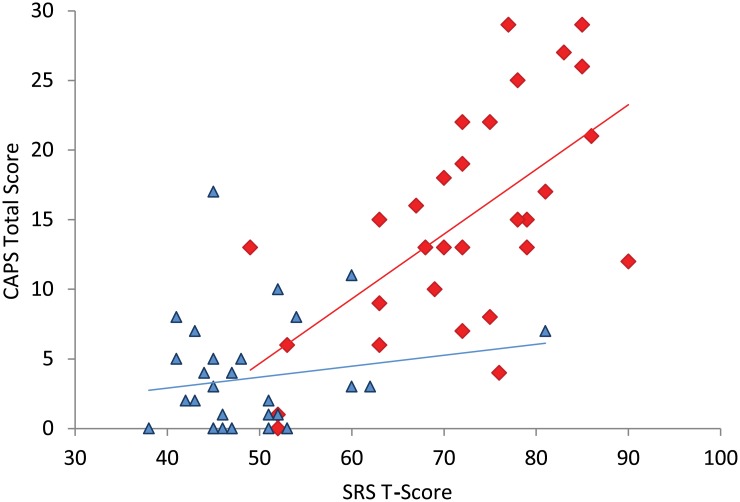
Scatterplot showing SRS T-scores and CAPS total scores. Participants with ASC are indicated by red diamonds. Participants without ASC are indicated by blue triangles. Linear regression lines for the two groups are also shown.

To investigate whether or not there was a relationship between SRS T-scores and anomalous perception a series of correlation analyses were carried out using the following variables for each group: SRS T-score; CAPS total score; distress; intrusion; frequency. SRS T-score did not correlate with any of these variables in the participants without ASC (all ρ <.22). In the participants with ASC all of the CAPS variables were significantly related to SRS T-score (N = 30, CAPS total score, ρ = .574, p <.001; distress, ρ = .584, p <.001; intrusion, ρ = .539, p <.001; frequency, ρ = .647, p <.001). A scatterplot showing SRS T-scores and CAPS total scores is presented in Fig 3.

## Discussion

**Fig 4:**
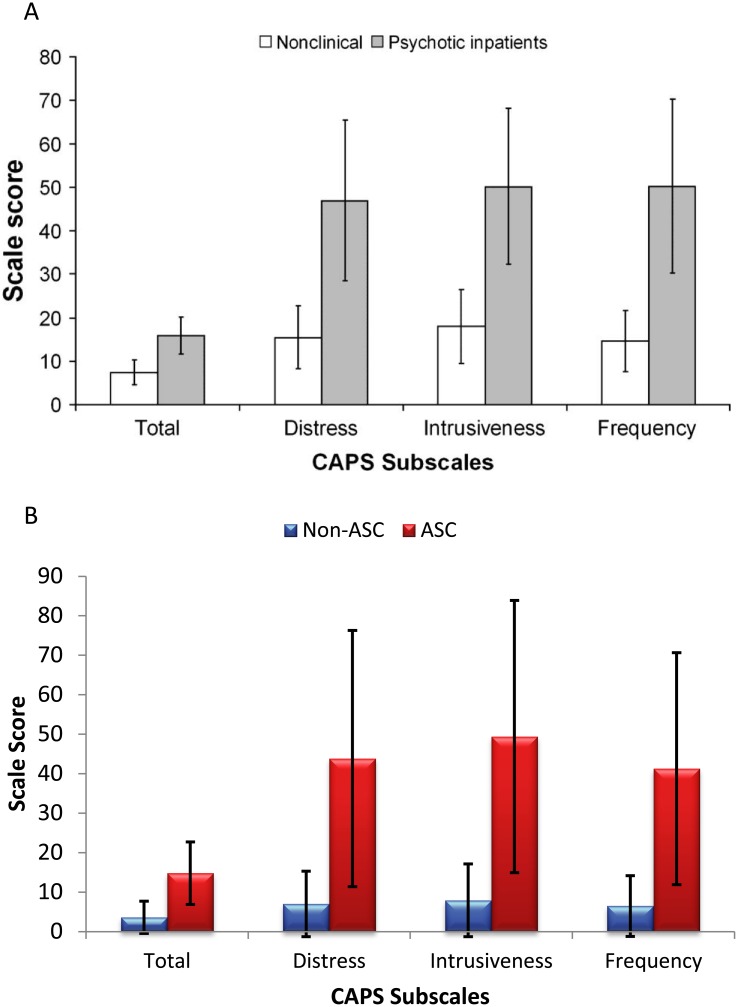
A comparison of the data collected here and previous data published by Bell et al. 2006. (A) Raw CAPS scores (means and standard deviations) from 337 neurotypical participants and 20 psychotic inpatients. Reprinted from Bell et al. (2006), Schizophrenia Bulletin. 2006; 32(2), 366–77, Oxford University Press, under a CC BY license, with permission from Professor Peter Halligan, original copyright 2005. (B) Raw CAPS scores (means and standard deviations) from 30 neurotypical participants and 30 participants with ASC (this study).

Here we used the CAPS to measure anomalous perception in ASC and found that adults with ASC report significantly higher levels of anomalous perception than adults without ASC. In addition, we found that levels of distress associated with anomalous perception are higher in individuals with ASC than in individuals without ASC and that anomalous perceptual experiences are more intrusive in those with ASC. In the paper which first described the CAPS [11] data were obtained both from a general population sample and from a sample of twenty psychotic inpatients. In Fig 4 we show the data from this initial publication [11] alongside the data collected here. For ease of comparison with the original study [11], in this figure we show raw data rather than scaled scores or percentages. It is clear from Fig 4 that the level of anomalous perception reported here by the individuals with ASC was as high as the level reported by hospitalised individuals who were experiencing acute psychotic symptoms (reported in [11]). In addition, levels of distress, intrusion, and frequency of anomalous perception reported by this sample of adults with ASC were very similar to those of the hospitalised sample. While we predicted that anomalous perceptual experience may be increased in individuals with ASC compared to neurotypical controls, the magnitude of this increase, and its similarity to individuals with psychosis was striking, especially as previous or current diagnosis of any psychosis spectrum disorder was an exclusion criterion of the study.

Two important implications emerge from this work: (i) some adults with ASC experience high levels of anomalous perception, and (ii) the experience of anomalous perception may be associated with significant distress in those with ASC. These findings highlight the need for full psychiatric assessment during clinical evaluation for ASC, c.f. [15]. Clinical work-up for individuals with ASC does not typically involve asking whether or not a participant experiences anomalous perception and patients may not spontaneously volunteer this information. Therefore, identifying whether anomalous perception is experienced by patients with ASC should be considered as part of clinical assessment. Here, 28 out of 30 individuals with ASC obtained CAPS scores higher than the mean score reported in the non-ASC group, suggesting that anomalous perception may be experienced by the majority of high functioning adults with ASC.

Classification and diagnostic systems such as the DSM and the ICD do not permit dual diagnoses for ASC and psychiatric conditions such as schizophrenia, bipolar disorder or psychotic disorder. Nevertheless, the data presented here adds to a small, but growing body of literature which highlights a degree of symptom overlap between ASC and psychotic disorder. For example, using a modelling approach, Rzhetsky et al. [16] demonstrated that autism, bipolar disorder and schizophrenia have significant genetic overlap, and suggested that is there is likely a genetic variation that predisposes an individual to all three conditions. Epidemiological studies also suggest that symptoms of psychosis occur more frequently in adults with ASC than in their neurotypical counterparts [15, 17–19]. This suggests the need for a more individual approach to diagnosis and therapy for individuals with ASC, and highlights limitations in current classification systems which perhaps do not capture, and to some extent deny the existence of, the full range of symptoms associated with ASC.

There are a number of potential pathways that may lead to anomalous perception in ASC. At a biological level, reduced NMDA-receptor function has been implicated in the pathophysiology of both autism and schizophrenia [20]. This, in turn, may lead to disruption of the balance of neural excitation and inhibition, which may lead to alteration of perceptual experience [21], and to anomalous perception in a number of different clinical conditions [22]. Previous work has shown that individuals who report increased incidence of pattern-glare—a phenomenon that has been associated with elevated cortical excitability—also experience increased levels of anomalous perception [8]. This is intriguing in-light of a growing collection of studies that suggest atypical excitation:inhibition balance in ASC (see [23] for a review).

Another potential route to anomalous perception is previous experience of traumatic events. For example, repeated experience of childhood bullying can lead to subclinical levels of psychotic experience in the general population [24]. High levels of bullying have been reported in children with ASC [25, 26], and it is possible that one of the consequences of such traumatic childhood experience may be increased incidence of anomalous perception (c.f. [27]). These suggestions remain speculative at present as the data presented here are not comprehensive enough to be able to identify exactly why anomalous perception occurs more frequently in adults with ASC than in those without ASC. Nevertheless, the fact the data clearly show increased incidence of anomalous perception in individuals with ASC highlights that this area clearly warrants further investigation.

As can be seen in Fig 4, the average CAPS score in our non-ASC sample (3.9) was lower than the average CAPS score found in the larger group of neurotypical individuals (7.3) reported by Bell et al. [11]. One reason for this could be that the sample recruited here was older than the sample recruited by Bell et al. Here, the mean age of the neurotypical participants was 35, whereas in Bell et al.’s sample the mean age of the neurotypical sample was 21.6. Previous work has shown that younger adults tend to show higher psychosis ratings than older adults [28], therefore our fining of lower CAPS scores in our neurotypical sample when compared with that presented by Bell et al. is not surprising.

There are a number of limitations to this study. For example, it is possible that other variables which were not measured here may mediate the association between ASC and anomalous perception. For example, in addition to finding a significant correlation between autistic traits and anomalous perception, Horder et al. found that self-reported level of anxiety was also correlated with CAPS scores [5]. As we did not measure anxiety we cannot investigate any potentially mediating role in the relationship between ASC and anomalous perception. Furthermore, all of the variables reported here were obtained from self-reported questionnaires, and we cannot rule out the concern that common-method bias may be driving the relationship between SRS scores and CAPS scores in the participants with ASC. Future studies should employ different techniques including face to face interviewing, for example using an instrument such as the Structured Interview for Assessing Perceptual Anomalies [29], and/or qualitative techniques to further probe the extent to which anomalous perception occurs in ASC, and the ways in which it affects daily living in adults with ASC. It is also important to note that the current report describes data obtained from adults with ASC who live independently and have IQs in the normal range. It is not yet known whether anomalous perception occurs in lower-functioning adults with ASC, and/or whether anomalous perception occurs in children with ASC.

To conclude, here we show that adults with ASC experience significantly higher levels of anomalous perception compared to a well-matched neurotypical control group. 93% of the participants with ASC reported higher levels of anomalous perceptual experience than the mean level reported by the participants without ASC. Strikingly, the extent to which anomalous perception was reported in ASC, and the associated levels of distress and distraction are as high in individuals with ASC as in hospital in-patients who are experiencing a psychotic episode. This finding has important implications for the clinical care of adults with ASC. Establishing whether or not an individual with ASC is experiencing distress and distraction associated with anomalous perception is an important part of clinical practice, so that appropriate psychological therapeutic approaches can be taken to minimise this associated distress.
